# Carbon-Nanotube-Coated Surface Electrodes for Cortical Recordings In Vivo

**DOI:** 10.3390/nano11041029

**Published:** 2021-04-17

**Authors:** Katharina Foremny, Wiebke S. Konerding, Ailke Behrens, Peter Baumhoff, Ulrich P. Froriep, Andrej Kral, Theodor Doll

**Affiliations:** 1 Institute of AudioNeuroTechnology and Department of Experimental Otology, ENT Clinic, Hannover Medical School, 30625 Hannover, Germany; konerding.wiebke@mh-hannover.de (W.S.K.); behrens.ailke@mh-hannover.de (A.B.); baumhoff.peter@mh-hannover.de (P.B.); kral.andrej@mh-hannvoer.de (A.K.); doll.theodor@mh-hannover.de (T.D.); 2 Division of Translational Biomedical Engineering, Fraunhofer Institute for Toxicology and Experimental Medicine ITEM, 30625 Hannover, Germany; ulrich.froriep@item.fraunhofer.de

**Keywords:** carbon nanotubes, etching, flexible electrode, in vivo, silicone composite

## Abstract

Current developments of electrodes for neural recordings address the need of biomedical research and applications for high spatial acuity in electrophysiological recordings. One approach is the usage of novel materials to overcome electrochemical constraints of state-of-the-art metal contacts. Promising materials are carbon nanotubes (CNTs), as they are well suited for neural interfacing. The CNTs increase the effective contact surface area to decrease high impedances while keeping minimal contact diameters. However, to prevent toxic dissolving of CNTs, an appropriate surface coating is required. In this study, we tested flexible surface electrocorticographic (ECoG) electrodes, coated with a CNT-silicone rubber composite. First, we describe the outcome of surface etching, which exposes the contact nanostructure while anchoring the CNTs. Subsequently, the ECoG electrodes were used for acute in vivo recordings of auditory evoked potentials from the guinea pig auditory cortex. Both the impedances and the signal-to-noise ratios of coated contacts were similar to uncoated gold contacts. This novel approach for a safe application of CNTs, embedded in a surface etched silicone rubber, showed promising results but did not lead to improvements during acute recordings.

## 1. Introduction

### 1.1. The Requirement of Small Electrode Contact Diameters

Current approaches in electrophysiology, both in basic research and biomedicine, have an increased need for high spatial acuity and long-term stability for chronic recording/stimulation. There, recording and/or stimulation is performed simultaneously at several neuronal sites utilizing multi-electrode arrays to assess the functional link between cortical areas [1], the spatial delimitation of pathological brain function as observed in epilepsy [2], and direct electrical stimulation of nerves, for example, to restore hearing in deafened patients [3]. An important step is therefore the reduction of electrode contact size while maintaining efficient electrochemical function, ideally combined with direct interfacing of the electrodes with the neurons [4]. In current metal electrodes the potential for contact diameter reduction is limited: they can cause irreversible electrochemical reactions during stimulation and the elevated impedances lead to low signal-to-noise ratios (SNR) during recording [4]. To overcome these limitations, the effective contact area has to be enlarged by increasing the surface area while keeping the contact diameter small [5]. This can be achieved via micro- and nanostructured platinum-iridium contacts [6], iridium-oxide modifications with superior electrophysiological maximum charge limits [7], conducting polymers like polypyrrole and PEDOT [8], or carbon electrodes based on graphite/graphene as well as carbon nanotubes (CNTs) in numerous variants and oxidation states [9,10,11,12]. The CNT nanostructure leads to significantly enlarged effective contact surface areas due to the resulting rough structure, which enables reduction of the contact diameter while retaining a high effective area [13].

### 1.2. Applications of CNTs to Improve Effective Electrode Contact Surface Areas

CNT electrodes provide several advantages over metal electrodes, as they are chemically inert capacitive conductors with good electrical conductivity (for review see [4,13]). This is especially beneficial for electrical stimulation, as it enables high current density pulses without causing irreversible faradaic reactions, thus preventing tissue damage [13]. Additionally, CNTs are well suited for neuronal interfacing, as they enable a direct connection between the neurites and the electrodes [4]. This electrode-tissue interface may further improve during long-term recordings/stimulations, as CNTs have been described to facilitate cell attachment [14,15] and permit neurite outgrowth from attached neurons [16]. Tissue-penetrating CNT microelectrodes for neuronal recordings increase signal sensitivity and decrease neuronal noise [17].

Many studies use CNTs that are directly grown on the substrate (e.g., [10,18]), which would not be possible using state-of-the-art flexible surface electrocorticographic (ECoG) electrodes due to the high synthesizing temperatures. An alternative approach is to combine CNTs with other contact materials, e.g., gold or polypyrrole [5], or to embed CNTs into materials by first growing them on an electrode substrate and then coating this composition with e.g., Parylene-C [19]. Still, most of the current advances in CNT-coated electrodes involve rigid initial materials, which are not ideally suited for chronic in vivo applications, such as recordings from the cortical surface [13]. To overcome the constraint of a rigid substrate, but still obtain the benefits of CNT-structured surface areas, CNT-coatings of flexible electrode substrates, such as Parylene-C or polydimethyl-siloxane (PDMS, silicone rubber), were introduced [19,20,21]. One possibility is direct CNT growth into silicon and thin-film processing to uncover the electrode area [10,19]. Burblies et al. [22] applied CNTs in an aqueous solution onto platinum substrates, which can withstand the regular handling during standard material evaluation tests or a week-long storage in cell culture media. Yet, if the surface is touched or rubbed along, e.g., tissue, the coating may be removed. This is disadvantageous, since CNT release from the electrode into the human body has to be prevented due to their high bio-toxicity [23]. The approach also applied in the present study is the embedding of CNTs in the respective polymer [19,21]. This eliminates the risk of CNT detachment from the electrode. Thus, it substantially reduces the risk of CNT release into surrounding tissue and fluids. However, when embedding CNTs, the advantage of the structured surface area is highly decreased [24]. Thus, the insulating shield on top of the CNTs (e.g., 150 µm in [21]) has to be removed to expose the required nanostructure of the CNT material.

### 1.3. The CNT-Coated Surface Electrode

We previously described a technique for CNT-coating of electrodes by applying a CNT-silicone rubber composite onto metal electrode contacts and a subsequent wet or plasma (dry) etching of the composite surface [25]. The remaining thin PDMS layer allowed for electron tunneling without releasing CNTs into the tissue due to safe anchoring of the CNTs in the electrode substrate [26]. The exposed CNT nanostructure has already been shown to enable attachment of neurites [24]. Using state-of-the-art thin-film metal electrodes as substrates, conventional recording systems can be used and the electrode performance can directly be compared to that of uncoated metal contacts [27]. In the present study, we assessed whether the method of etched CNT-silicone composite coating of state-of-the art metal electrodes results in signal recording quality comparable to state-of-the-art ECoG electrodes. Based on the proposed advantages for electrode-tissue interfacing and via reduced impedances, we expected improved signal-to-noise ratios as compared to uncoated surface electrodes. Therefore, we analyzed the efficiency of different etching methods to reduce the impedance of the CNT-coated contacts and tested the signal quality during acute in vivo recordings of auditory evoked potentials from the primary auditory cortex of the guinea pig. We show that etching of CNT-silicone rubber surfaces on ECoG contacts was performed successfully. These electrodes allowed for impedance and recording results that kept up with the state-of-the-art gold contacts, leading to a possible signal recording benefit during future chronic applications.

## 2. Materials and Methods

### 2.1. CNT-Coated ECoC Electrodes

**Figure 1:**
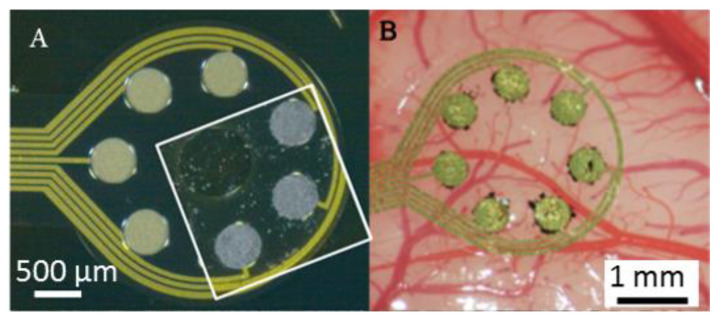
Representative photographs of the ECoG electrode grid with gold contacts and CNT-silicone rubber coated contacts (white frame) (**A**). Representative photographs of the ECoG electrode with CNT-coated gold contacts positioned subdurally on the guinea pig auditory cortex (**B**).

The ECoG electrodes (Blackrock Microsystems Europe) were composed of a flexible Parylene-C substrate (20 µm thick) with 7 circular gold contacts (500 µm in diameter, 0.2 mm^2^) and a circumferential gold reference (Figure 1A). The electrode substrate is highly flexible (Young’s modulus ~4GPa [28]), which enables a more intimate contact to the brain tissue (Figure 1B). The 7 gold contacts were coated with a CNT-silicone rubber composite. The processing steps have already been reported in detail previously [24,26]. In brief, 5 or 10 *w*/*w*% of multi-walled CNTs (NCT7000, Nanocyl, Sambreville, Belgium: 9.5 nm diameter, 1.5 µm length) were immersed in a room temperature vulcanized two component polydimethyl-siloxane (PDMS) Sylgard^®^ 184 (Dow Corning, Midland, MI, USA). The curing agent was added in a ratio of 1:10, and subsequently the material was spread out onto the gold contacts and cured (4 h at 70 °C).

For the in vivo experiments, the previously evaluated composites with 5 *w*/*w*% CNTs in PDMS were used by applying the compound to the contacts using glass blades [25]. For correlation with the electrophysiological results, the impedances of each recording electrode were measured (1 kHz, 1 V, system: Alpha Omega Engineering). Additional impedance measurements were performed with 10 *w*/*w*% CNT compounds after improvement of the processing steps. The higher CNT proportion was possible due to a finer dispersion of the material using a 3-roll mill (50I, EXAKT, Norderstedt, Germany). Default settings were used, resulting in a gap size of 15/7 (~150 µm/70 µm) and a velocity of 8 (~478 rpm; one pass). Furthermore, by using a photoresist molding procedure as introduced in [25], the composite thickness (30 µm) and spread on the electrode contacts were more accurate.

For both in vivo recordings and impedance measurements, we compared different etching methods for reducing the insulating silicon rubber shield on the CNTs. The CNT-silicone rubber surface for the electrode contact was etched using either wet or dry etching methods (for details see [25]). We used two wet etchants: tetra-n-butylammonium fluoride (TBAF) with either N-Methyl-2-pyrrolidone (NMP) or dimethylformamide (DMF) as solvent (ratio 1:3), and one dry etching method, with a combination of tetrafluoromethane (CF_4_) and oxygen (O_2_; ratio 3:1). We additionally compared different etching times for wet (1, 5 or 10 min) and dry etching (10, 30 or 90 min). To quantify the etching success, we performed scanning electron microscopy (SEM, [24]) to measure the diameter of visible CNTs. The residual silicone rubber shield can be calculated from the measured diameter and the known diameter of the used CNTs. Impedances were measured at the biologically relevant frequency of 1 kHz ([5]; platinum reference electrode; 0.9% saline solution). The impedance values were compared to those of an uncoated gold electrode and an un-etched CNT-silicone rubber surface (CNT-control). Furthermore, the impedance values were correlated with the thickness of the residual silicone rubber shield.

### 2.2. In Vivo Recordings

The acute in vivo tests were performed in an anesthetized Dunkin–Hartley (albino) guinea pig. All procedures were in accordance with the German and European Union guidelines for animal welfare (ETS 123, Directive 2010/63/EU) and were approved by the German state authority (Lower Saxony state office for consumer protection and food safety, LAVES). An appropriate anesthesia level for cortical recordings was maintained by ≤0.7% isoflurane in 1:1 O_2_: air and was surveyed by regular monitoring of vital parameters and paw-withdrawal or corneal reflexes. Normal hearing was confirmed by auditory brainstem responses (click response threshold ≤ 35 dB sound pressure level (dB_SPL_) peak equivalent). For details on animal preparation and surgery see [29]. In brief, to access the cortex, the skull was exposed and a head-holder (custom made, stainless steel fixation-rod) was secured to the bone anterior to suture-point Bregma, using 3 bone screws (Ø 0.85 mm, Fine Science Tools GmbH, Heidelberg, Germany) covered with dental acrylic cement (Paladur, Heraeus Kulzer GmbH, Dormagen, Germany). As recording reference, a silver ball electrode covered in salt-free electrode gel (Spectra 360, Parker Laboratories INC., New Jersey, USA) was placed through a hole ~1 mm rostral from Bregma onto the dura mater.

**Figure 2:**
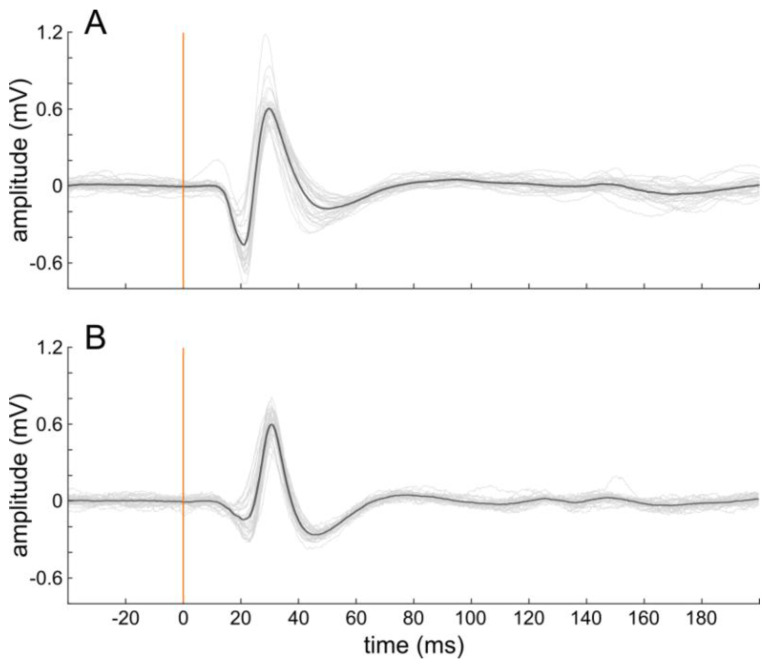
Representative cortical auditory evoked responses, showing high recording quality for both an untreated gold contact (**A**) and a CF_4_/O_2_-etched CNT-coated contact (**B**). Given are 30 single repetitions (grey) and the resulting average signal (black) in response to a 60 dB_SPL_ broadband stimulus. The vertical line indicates the stimulus onset (100 ms white noise).

**Figure 3:**
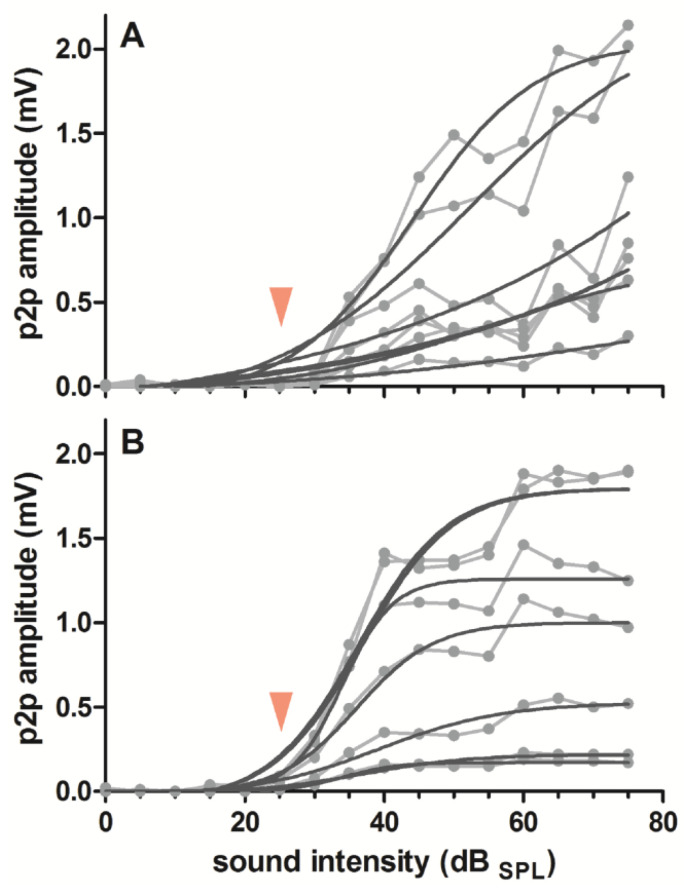
Threshold determination via input–output functions revealed similar thresholds (25 dB_SPL_, arrow head) for the untreated gold electrode (**A**) and a representative CF_4_/O_2_-etched CNT-coated ECoG electrode (**B**) with slight differences between electrode contacts/recording positions (*N* = 7). Given are the input–output functions for the LFP p2p amplitude over different stimulus intensities (noise burst: 0–70 dB_SPL_). Individual data (grey dots) are connected by lines (grey), and the resulting sigmoidal fit is indicted (black; goodness of fit: A: r^2^ ≥ 0.813; B: r^2^ ≥ 0.958).

A unilateral craniotomy (~5 mm × 5 mm) was performed, centered at ~2.5 mm caudally from Bregma and 7.3 mm laterally from the midline to expose the primary auditory cortex. To describe the quality of neuronal recordings with the CNT-coated surface electrodes, we assessed the response amplitude to broadband noise stimuli and the signal-to-noise ratio (SNR) during an acute recording (examples see Figure 2). To generate the input–output functions for analyses relative to the response threshold (see below), we presented 100 ms white noise bursts (10 ms cosine ramps) in seventeen, 5 dB steps (0–80 dB_SPL_). The neuronal signals were amplified (8000×) and acquired through a multichannel recording system (Lynx-8 amplifier system, butterworth filter: 1 Hz–9 kHz, rolloff: 12 dB per octave, Neuralynx, Bozeman, MT, USA) and stored through a custom-built recording setup and the corresponding stimulation and data acquisition software (AudiologyLab, Otoconsult, Frankfurt, Germany) at a sampling rate of 25 kHz using a 32-channel MIO card (NI-6259 National Instruments, Austin, USA). Neuronal signals were averaged over 30 repetitions to analyze slow local field potentials (LFP; resampled at 2 kHz using Matlab). The response strength was assessed as LFP peak-to-peak (p2p) amplitudes in a time window of 0–200 ms after stimulus onset. The p2p amplitudes were baseline-corrected by subtraction of the respective background p2p amplitudes (50 ms before stimulus onset). The SNR was calculated as the response p2p amplitude, relative to the average background p2p amplitude in dB. To account for minor LFP threshold differences between recording positions, we analyzed the response amplitudes and the SNR at a defined stimulus level of 30 dB above threshold. The threshold level was assessed for each contact separately by fitting a sigmoidal function, defining the threshold as 10 % of the calculated maximal amplitude (Figure 3, e.g., [5]). Sometimes, the highest sound pressure level (80 dB_SPL_) induced lower response amplitude than the second highest (75 dB_SPL_), most probably due to the middle ear muscle reflex at high sound intensities [29]. Thus, the fitting was performed following exclusion of the 80 dB response amplitude.

### 2.3. Statistics

The impedance data were compared using nonparametric test procedures to account for small sample sizes. When the global analysis (Kruskal–Wallis test) revealed a significant difference between groups, the groups were further compared via a Dunn’s post-hoc test. A spearman correlation was used to assess the potential relation between impedance and residual rubber shield thickness. For the cortical recordings, normal distribution of the data was confirmed via the Kolmogorov–Smirnov test (*p* > 0.05). Subsequently, one-way ANOVAs (multiple groups) or independent t-tests (two groups) were performed. The level of significance was set to 5%.

## 3. Results

**Figure 4:**
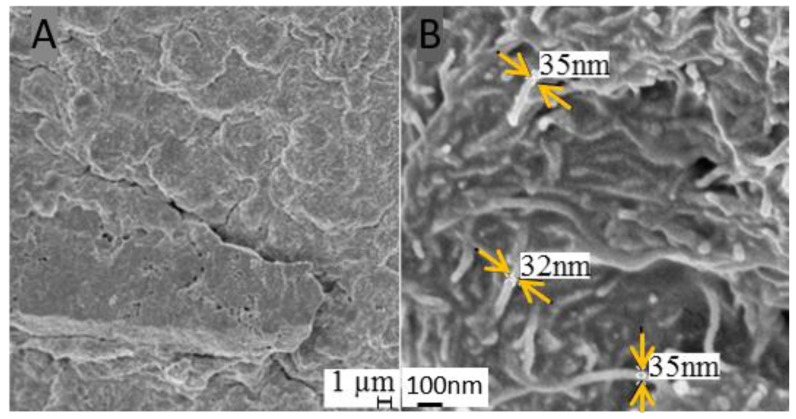
The SEM image of a CNT-silicone rubber surface (**A**), and the enhanced etched surface including diameters (between arrows) of representative coated CNTs (**B**).

To investigate the suitability of CNT-silicone rubber coated ECoG electrodes for future chronic applications, we started off by assessing the impedances and recording performance of these electrodes in comparison to state-of-the-art gold electrodes. We successfully coated the gold electrodes with CNT-silicone rubber (see Section 2.1.). Etching was successfully performed resulting in a nano-pattern of embedded CNTs as revealed via scanning electron microscopy (Figure 4).

### 3.1. Impedances

**Figure 5:**
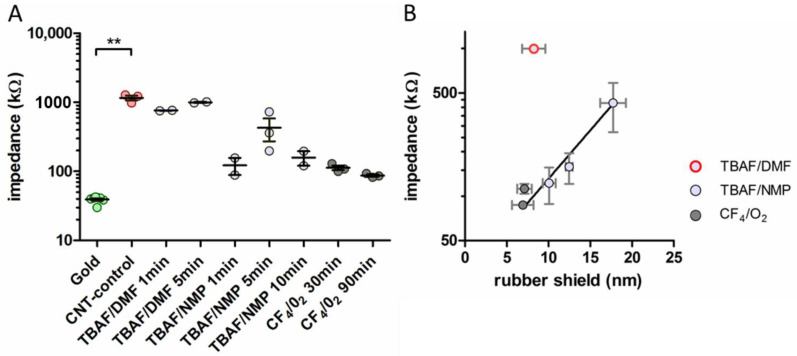
(**A**) Impedances of etched CNT-coated contacts were similar to those of the untreated gold electrode. Given are individual data (dots) and mean with SEM (line) for each of the different ECoG electrodes impedance values. Values are given in logarithmic scale. Kruskal–Wallis test with Dunn’s post-hoc test: ** *p* < 0.01. (**B**) Correlation between impedance and residual silicone rubber shield on the CNTs at the contact surface. Depicted are values in a linear-log graph with mean (dots) and SEM (line) for the different wet and dry etching methods. Excluding the TBAF/DMF probes revealed an exponential association of the (linear) data (black line).

The un-etched electrode coated with CNT-silicone rubber (CNT-control) showed significantly higher impedances (mean: 1160.02 kΩ) in comparison to the untreated gold contacts (mean: 39.27 kΩ; Kruskal–Wallis test: K = 24.90, *p* = 0.002, *n* = 9 groups; Dunn’s post-hoc test: *p* < 0.01). Most etched materials showed smaller impedances compared to the un-etched CNT-control electrode (Figure 5A, Dunn’s post-hoc test: *p* > 0.05); however, TBAF/DMF had high values (min. 753.42 kΩ) similar to the CNT-control. The lowest impedances were found for the CF_4_/O_2_-etched probes (min: 82.14 kΩ, max: 128.95 kΩ), which were still higher than the untreated gold contacts (max: 42.62 kΩ). When combining all data, the impedances of the etched electrode contacts were not significantly correlated with the thickness of the residual, insulating silicone rubber shield (Spearman correlation: *p* = 0.175, r^2^ = 0.432, *n* = 6; Figure 5B). However, when excluding the TBAF/DMF-treated probe that showed impedance values comparable to the insulated CNT-control (Figure 5B), the correlation was highly significant (*p* = 0.017, r^2^ = 1.000) with an exponential relation between impedance and residual rubber shield thickness (y = 28.97*e0.151x, r^2^ = 0.591; Figure 5B).

### 3.2. Response Amplitude and Signal-to-Noise Ratio

**Figure 6:**
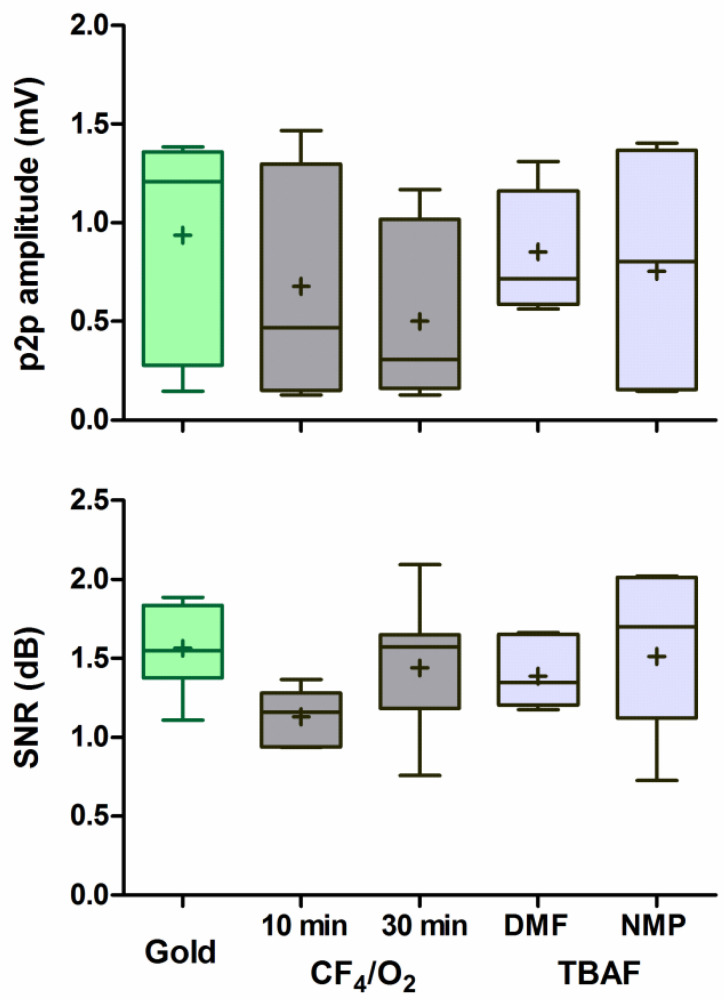
The response amplitude (peak to peak, p2p) and SNR of etched CNT-coated contacts was similar to the untreated gold contacts. Amplitudes and SNR at 30 dB above threshold are given as box-plots (median and interquartile range) with min and max values (whiskers) and mean (cross; *N* = 7).

Control recordings with the untreated gold-electrode were performed twice: once preceding and once succeeding the recordings with the etched CNT-electrodes. As the recordings at the two time-points (and slightly different recording positions) did not differ significantly (p2p amplitude: *p* = 0.212, t = 1.486, df = 4; SNR: *p* = 0.505, t = 0.731, df = 4), we combined the values for further analyses. Recordings were possible with all 7 electrode contacts with low variability between single presentations (Figure 2). Both the LFP response amplitude and the SNR did not differ between the different ECoG electrodes, independent of CNT-coating, or etching method (1-way ANOVA: p2p amplitude: *p* = 0.528, F = 0.811, *n* = 5 groups; SNR: *p* = 0.196, F = 1.619, *n* = 5; Figure 6). Qualitatively, the two wet-etched CNT-coated ECoG electrodes showed on average higher amplitudes and SNR-values than the CF_4_/O_2_-etched ECoGs and thus were more similar to the untreated gold electrode. The impedance values of the etching methods did not account for these differences, as the cortical LFP responses did not correlate with the impedances of the respective contacts, both for the p2p amplitude (Pearson correlation: *p* = 0.725, r^2^ = 0.004) and for the SNR (*p* = 0.375, r^2^ = 0.245). During these acute recordings, no biocompatibility issues or adverse events were observed.

## 4. Discussion

In this study, we demonstrated that etched CNT-coated ECoG electrodes exhibited impedances in the range relevant for neurophysiological application. The study confirmed their usability for brain recordings. The CNT-silicone rubber composite was successfully applied to flexible ECoG electrode contacts without compromising their advantages for handling and recording. The initial insulation of the gold contacts by coating with CNT-silicone rubber was successfully reduced by both wet and dry etching. Thereby, dry (plasma) etching was most effective. This method allows for safe anchoring of the CNTs in the substrate while exposing the CNT nanostructure.

### 4.1. Impedance

Several studies have shown that coating with pure CNTs can significantly reduce the impedance of an electrode contact (e.g., [5,17,19]). Therefore, our initial expectation was a reduced impedance relative to the uncoated gold contacts. However, in our study, even the most efficient surface etching method (CF_4_/O_2_-etching for 90 min) led to slightly higher impedances than for the control gold ECoG electrode. Exemplary data of the impedance spectroscopy of the same electrodes have already been published by our group [25], demonstrating similar frequency characteristics as for the gold electrodes. In this study, we therefore compared impedances at the biologically relevant frequency of 1 kHz only. The increase in impedance was positively correlated with the remaining silicone rubber shield on the CNTs, as derived from SEM pictures [24,26] of electrodes produced and etched in the same passage. The exponential relationship between residual rubber shield and impedance fits to the exponential relation between conductor-conductor distance and the electron tunneling effect [30]. Only the etching via TBAF + DMF led to much higher impedances than assumed based on this relationship. We propose that this is due to a relatively large variation in etching success [24] as well as non-uniform dispersion of CNTs in the silicone rubber [31]. Thus, both the manufacturing and the etching have to be further standardized to result in a more uniform reduction of the PDMS layer on the CNTs. Another limitation for impedance-reduction is the rubber shield of the CNT-composite facing the gold contact. This cannot be reduced further with our surface etching methods. Thus, the enlarged effective surface area has to compensate for this effect first, before impedances can be reduced below the control level. However, to achieve impedances as low as the uncoated gold contact, the insulating shield would have to be reduced to less than 2.2 nm (based on the exponential relationship). It has been shown that due to CH-π interactions, the silicone rubber layers directly in contact with the CNTs cannot be easily removed [31], which reduces the chance of further reduction of the impendences. Furthermore, applying our etching techniques with longer exposure time (not shown here) did not further remove the silicone rubber but rather detached whole fragments of the material. Therefore, a further reduction of silicone rubber bears the risk of freely moving CNTs, which should be avoided due to bio-toxicity issues [23]. Even though the impedance could not be reduced below that of a gold electrode, the etching of the CNT-silicone rubber re-established the nanostructure of CNTs. A previous investigation with the etched CNT-silicone rubber [24] showed that neurites grow on this nanostructured surface. This may lead to better integration of the electrode into the neuronal tissue. Thus, the benefit of this electrode might increase during chronic applications via promoting a direct contact between outgrowing neurites and the electrode nanostructure.

### 4.2. Response Amplitude and Signal-to-Noise Ratio

The impedance of the etched CNT-coated electrode contacts was on average 90 kΩ. This was reasonably low for neuronal in vivo recordings and, as expected, we successfully recorded auditory evoked potentials from all seven contacts in all CNT-coated electrodes. Thereby, the CNT-coated ECoG electrodes showed similar performance as the untreated gold electrode. Some variability in the amplitude/threshold and form of the neural responses was observed (e.g., Figure 3). These variations are unlikely to be explained by the electrochemical impedance spectrum, as the spectrum of the etched CNT-coated gold contacts runs in parallel to the one of an untreated gold contact up to about 10 kHz [25]. This frequency is above the frequency range of LFPs (<100 Hz [27]). Thus, it is likely that the differences observed in the present study were rather to be explained by slight changes in recording position. The variability of the 30 repetitions recorded at each contact was, however, small (narrow spread around the mean; Figure 2), confirming the low background noise and high SNR in the recordings. Although, depending on the application, reduced impedances can be preferential for recording with high signal quality and SNR [13], recent studies suggest that, at least for LFP recording, the influence of impedance values and electrode contact geometry is negligible [32]. This was proposed to hold for the common range of conventional recording electrodes of 10 kΩ to 1 MΩ. This finding fits well with our observation that the impedances of our electrodes were uncorrelated to the signal amplitude or SNR recorded from the brain surface. As we focused here on local field potentials, we cannot rule out that reduction in impedance or a higher effective surface area will positively act on other electrophysiological measures, such as improving the SNR of action potential related multi-unit activity [27].

### 4.3. Outlook

The CNT-composite assessed here for its feasibility for neural recordings is currently under investigation in a short-term biocompatibility study in vivo. This will reveal the materials suitability regarding tissue reaction and immune responses. Besides the already implemented optimizations for the dispersion steps and the discussed need for optimal etching, further improvements can be applied for optimal electrode usage. These include the processing of the hydrophobic CNT-silicone rubber surface, to result in higher wettability, similar to what has already been successfully implemented in CNT-electrodes by UV-ozone modification [33]. These changes may increase the electrode-tissue coupling and may aid the intimate contact between the flexible and adhesive Parylene-C substrate and the brain tissue. Since our results showed that CNT-silicone-rubber-coated contacts were similar in performance to gold contacts while a neurite-attracting nanostructure on the electrode-nerve-interface was obtained, we expect benefits in future chronic recordings. In chronic neurite recording/stimulation applications of neurites with expected neurite outgrowth, e.g., the spiral ganglion neuron in the inner ear, the nanostructure resulting from the etching of the CNT-silicone rubber surface may allow for a direct connection between neurites and the electrode material.

## 5. Conclusions

In the present study, we showed that the coating of a flexible ECoG electrode with a CNT-silicone composite allowed for acute recordings of cortical responses to sensory stimulation in vivo. The impedances of the electrode contacts were adequately reduced by surface etching of the residual silicone rubber shield, exposing the CNT nanostructure. Although the impedances were slightly higher, the recording quality was similar to that of uncoated gold electrodes. Thus, the CNT-silicone rubber composite proposes good recording results that, in combination with the nanostructure of the interface, can be beneficial during chronic recoding due to direct coupling between multielectrode contacts and neural tissue.

## Data Availability

The data presented in this study are available on request from the corresponding author. The data are not publicly available due to the large size of the datasets generated and analyzed.
